# Comparison of outcomes between surgery and non-surgery after conversion therapy for advanced gastric cancer with unresectable factors: a systematic review and meta-analysis

**DOI:** 10.1186/s12876-025-03969-x

**Published:** 2025-05-14

**Authors:** Jiaheng Wu, Xuetian Du, Yiqiang He, Shulin Xian

**Affiliations:** 1 Department of Gastrointestinal Surgery, The First Affiliated Hospital of Guangxi Medical University, Nanning, Guangxi China; 2 Department of Gastrointestinal Surgery, Guangxi Nationalities Hospital, Guangxi, China

**Keywords:** Advanced gastric cancer, Conversion surgery, Overall survival, Disease-free survival

## Abstract

**Background:**

Advanced gastric cancer (AGC) with unresectable factors presents a significant treatment challenge. Conventional treatments such as systemic chemotherapy, radiotherapy, and immunotherapy can delay disease progression but often yield limited outcomes. For stage III-IV gastric cancer with unresectable factors, conversion therapy based on chemotherapy can achieve tumor downstaging, providing a subset of patients with the opportunity for curative surgery. However, the efficacy of multimodal approaches combining chemotherapy, with or without immunotherapy, and conversion surgery compared to chemotherapy alone remains controversial.

**Methods:**

We conducted a systematic review and meta-analysis of high-quality studies published between January 2014 and November 2024, assessing the role of surgery following conversion therapy in advanced gastric cancer. Relevant studies were retrieved from PubMed, Embase, and Web of Science databases. All included studies were observational; no randomized trials were available. Clinical data, including overall survival (OS), progression-free survival (PFS), objective response rate (ORR) and adverse event (AE) rates, were analyzed using RevMan 5.4.

**Results:**

Twelve observational cohort studies were included. Conversion surgery(CS) was associated with improved 1-year, 3-year, and 5-year OS rates (RR 0.38, 95% CI: 0.31–0.47; RR 0.64, 95% CI: 0.54–0.76; RR 0.77, 95% CI: 0.65–0.91, respectively) and increased 1-year and 3-year PFS rates (RR 0.57, 95% CI: 0.49–0.99; RR 0.67, 95% CI: 0.57–0.78, respectively). No significant difference in AE rates was observed between groups.

**Conclusions:**

Conversion surgery following chemotherapy in stage III-IV gastric cancer is associated with improved OS and PFS in observational studies. However, these findings may reflect inherent prognostic differences between groups, as surgery was only feasible for chemotherapy responders. Prospective trials are needed to validate causality.

**Supplementary Information:**

The online version contains supplementary material available at 10.1186/s12876-025-03969-x.

## Introduction

Gastric cancer (GC) ranks as the third leading cause of cancer-related deaths worldwide, representing a significant global health burden. In 2020, over one million new cases of gastric cancer were diagnosed globally, with approximately 769,000 deaths reported [1]. While early gastric cancer (EGC) is curable through endoscopic or surgical resection, most patients are diagnosed at advanced stages when curative surgery is no longer feasible. The majority of AGC cases are characterized by unresectable factors, such as extensive lymph node metastasis or distant metastasis [2]. Patients with AGC are often considered incurable. Conventional therapies, including systemic chemotherapy, radiotherapy, and immunotherapy, may prolong survival but rarely achieve long-term remission. No standardized chemotherapy regimen currently exists for unresectable gastric cancer, although regimens incorporating fluorouracil, platinum agents, irinotecan, capecitabine, paclitaxel, docetaxel, or S-1, with or without immunotherapy, have shown survival benefits [3–8]. Conversion therapy, defined as systemic chemotherapy aimed at downstaging tumors to render them resectable, offers new hope for some patients with unresectable stage III-IV gastric cancer [9, 10]. Successful conversion therapy enables curative surgery (conversion surgery, CS) and may improve long-term outcomes for AGC patients [2, 11]. Several studies have reported superior survival outcomes in stage III-IV gastric cancer patients undergoing conversion surgery compared to those receiving chemotherapy alone [12–14], suggesting that conversion surgery may be a viable curative option. However, the role of this multimodal approach remains contentious. This meta-analysis aims to systematically evaluate the efficacy and safety of conversion surgery versus chemotherapy alone in AGC patients with unresectable factors, providing robust evidence to guide clinical practice.

## Methods

We performed a comprehensive search of PubMed, Embase, and Web of Science databases to identify randomized controlled trials and non-randomized studies comparing outcomes of conversion surgery versus non-surgical management in AGC with unresectable factors. Key outcomes analyzed included overall survival (OS), progression-free survival (PFS), objective response rate (ORR), and adverse event (AE) rates. OS/PFS outcomes were calculated using mortality counts. Surviving patients were censored at the last follow-up. Data extraction and statistical analyses were conducted using RevMan 5.4 software. Relative risk (RR) and 95% confidence intervals (CIs) were calculated for all outcomes.

### Literature search strategy

The systematic review adhered to the PRISMA guidelines. Two independent researchers searched PubMed, Embase, and Web of Science databases up to November 2024. The search was limited to studies published in English within the last decade. Keywords included “surgery,” “operation,” “gastric cancer,” “gastric carcinoma,” “cancer of stomach,” and “chemotherapy.” Duplicates were removed, and references from retrieved articles were reviewed to identify additional relevant studies. Studies without available abstracts or full texts were excluded. For duplicate publications, the most recent version with extended follow-up or larger sample size was included.

### Inclusion criteria and primary outcome measures

This study aimed to evaluate the comparative efficacy of conversion surgery versus non-surgical treatment in advanced gastric cancer (AGC) patients with initially unresectable factors. The inclusion criteria followed the PICO-S framework as outlined below:

#### Participants (P)

Patients with unresectable AGC undergoing conversion therapy (chemotherapy with or without immunotherapy). Studies without a non-surgical control group or case reports were excluded.

#### Intervention (I) and Comparison (C)

Patients receiving conversion surgery after therapy were included in the intervention group, while those undergoing conversion therapy without surgery formed the control group.

#### Outcomes (O)

Primary endpoints included 1-year, 3-year, and 5-year OS rates and PFS rates. Secondary endpoints included response rates (complete response [CR] and partial response [PR]) and adverse events during conversion therapy (e.g., neutropenia, anemia, nausea, diarrhea, intestinal obstruction, liver dysfunction, renal dysfunction).

#### Study Design (S)

Only high-quality observational cohort studies were included.

### Literature search and identification

**Fig. 1:**
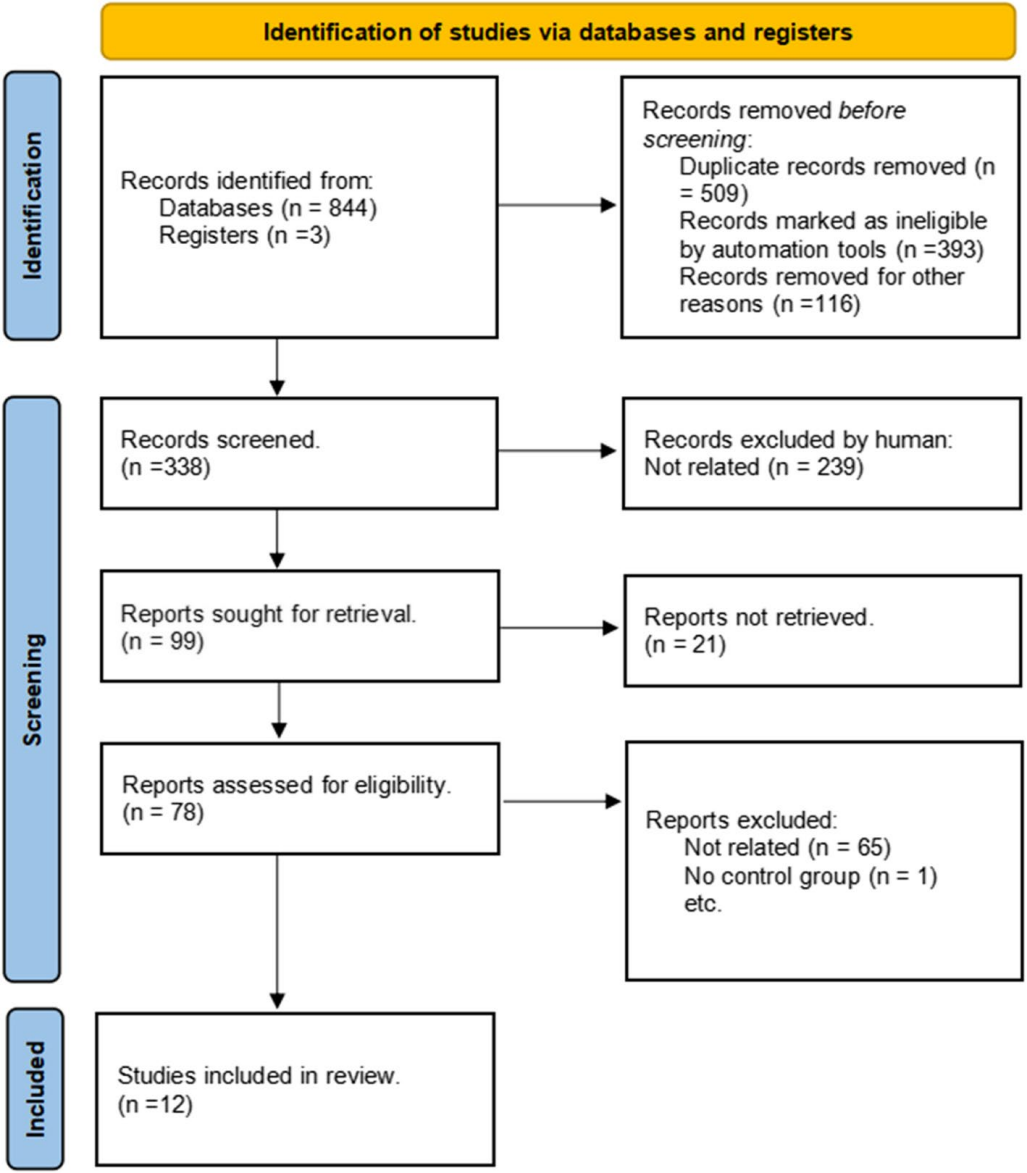
PRISMA flow diagram of present meta-analysis

Two independent researchers conducted a comprehensive search of PubMed, Embase, and Web of Science databases. To mitigate publication bias, we searched ClinicalTrials.gov, WHO ICTRP, and Grey for ongoing or unpublished studies. No additional eligible studies were identified. A total of 847 records were identified, with 338 articles remaining after duplicate removal. Abstract screening resulted in 78 studies being assessed for full-text eligibility. Of these, 66 were excluded for not meeting inclusion criteria. Ultimately, 12 studies [8, 7, 15–25] were included in the meta-analysis. The literature search and selection process are illustrated in a PRISMA flow diagram (Fig. 1).

### Risk of bias and study quality

The Newcastle–Ottawa Scale (NOS) was used by two researchers to assess the risk of bias in the included observational cohort studies. Studies scoring below seven points were excluded. Disagreements were resolved through discussion with a third researcher. The summarized quality assessment results are shown in Table 1.

**Table 1:** NOS score of study

Study	Representativeness of the exposed cohort	Selection of the non exposed cohort	Ascertainment of exposure	Demonstration that outcome of interest was not present at start of study	Comparability of cohorts on the basis of the design or analysis	Assessment of outcome	Was follow-up long enough for outcomes to occur	Adequacy of follow up of cohorts	Quality score
Wang 2014 [25]	1	1	1	1	1	1	1	1	8
Fukuchi 2015 [24]	1	1	1	1	1	1	1	1	8
Ito 2015 [23]	1	1	1	1	1	1	0	1	7
Kinoshita 2015 [22]	1	1	1	1	1	1	0	1	7
Li 2015 [21]	1	1	1	1	1	1	1	0	7
Sato 2017 [8]	1	1	1	1	1	0	1	1	7
Yamada 2016 [20]	1	1	1	1	1	1	1	1	8
Fukuchi 2018 [19]	1	1	1	1	1	1	1	1	8
Ohnuma 2021 [7]	1	1	1	1	1	0	1	1	7
Li 2021 [18]	1	1	1	1	1	1	1	1	8
Shinkai 2022 [16]	1	1	1	1	1	1	1	1	8
Liang 2023 [15]	1	1	1	1	1	1	1	1	8

### Data extraction

Two independent researchers extracted the following data: study characteristics (author, country, year of publication), tumor stage, intervention details, primary outcomes (OS and PFS rates), response rates (CR and PR), and adverse events (e.g., neutropenia, anemia, nausea, diarrhea, intestinal obstruction). The data extracted from each study is shown in Tables 2, 3 and 4.

**Table 2:** The characteristics and results of the interventions used in each study

Author + year	publish time	country	S	NS	total	age	male	female	metastatic sites	neoadjuvant chemotherapy regimen	postoperative chemotherapy regimen
Wang 2014 [25]	2014	China	28	20	48	63.5(35–77)	41	7	LN (+); PAN (+)	XELOX	XELOX
Fukuchi 2015 [24]	2015	Japan	40	111	151	66(31–79)	108	43	LN (+); P (+); H (+); CY (+)	SP; S-1 + Paclitaxel	S-1
Ito 2015 [23]	2015	Japan	14	56	70	64 ± 11.6	46	24	LN (+); P (+); L (+); B (+); S (+); CY (+)	S-1; SP; PX; Paclitaxel	N/A
Kinoshita 2015 [22]	2015	Japan	34	23	57	65(30–78)	38	19	PAN (+); P (+) H (+); LN (+); B (+)	DCS	N/A
Li 2015 [21]	2015	China	25	24	49	N/A	15	9	LN (+); L (+)	PX	PX
Sato 2017 [8]	2016	Japan	33	67	100	63(26–78)	71	29	LN (+); PAN (+); P (+); H (+); B (+); L (+); O (+)	DCS	DS
Yamada 2016 [20]	2016	Japan	44	28	72	68(22–87)	50	22	P (+); LN (+); H (+); CY (+)	SP	SP; S-1 + cisplatin + irinotecan
Fukuchi 2018 [19]	2018	Japan	31	63	94	69(31–82)	76	18	LN (+); P (+); L (+);	SP; DOS; SOX; XELOX + trastuzumab	N/A
Ohnuma 2021 [7]	2020	Japan	44	44	88	62.5(29–78)	61	27	LN (+); P (+); L (+); B (+)	DCS; DOS	S-1 + paclitaxel;irinotecan;SP;DCS
Li 2021 [18]	2021	China	40	40	80	N/A	46	34	LN (+); P (+)	SP; SOX	N/A
Shinkai 2022 [16]	2021	Germany	33	19	52	63(32–75)	33	19	LN (+); P (+)	Paclitaxel and S-1/cisplatin	S-1 + paclitaxel
Liang 2023 [15]	2023	China	42	94	136	57.5(25–80)	80	56	LN (+); PAN (+); P(+); L(+);O(+);CY(+)	(FOLFOX/FLOT/XELOX) + (Pembrolizumab/Sintilimab/Toripalimab/Nivolumab)	N/A

*N/A* not available, *SP* S-1 + cisplatin, *PX* paclitaxel + capecitabine, *DCS* docetaxel + cisplatin, + S-1, *DOS* docetaxel + oxaliplatin + S-1, *DS* docetaxel + S-1, *LN(* +*)* distant lymph node metastasis, *PAN(* +*)* para-aortic lymph nodes metastasis, *P(* +*)* peritoneum metastasis, *H(* +*)* Liver metastasis, *L(* +*)* lung metastasis, *CY(* +*)* positive peritoneal cytology, *O(* +*)* ovary metastasis, *B(* +*)* brain metastasis, *SP* S-1 + cisplatin, *PX* paclitaxel + capecitabine, *DCS* docetaxel + cisplatin + S-1, *DOS* docetaxel + oxaliplatin + S-1, *DS* docetaxel + S-1

**Table 3:** The characteristics and results of the interventions used in each study

Author + year	MF-U(month)	OS-1				OS-3				OS-5				PFS-1				PFS-3				PFS-5				CR				PR			
Group		S		NS		S		NS		S		NS		S		NS		S		NS		S		NS		S		NS		S		NS	
Wang 2014 [25]	12.4	8	28	12	20	19	28	15	20	N/A	N/A	N/A	N/A	13	28	15	20	15	28	20	20	N/A	N/A	N/A	N/A	0	28	2	20	20	28	1	20
Fukuchi 2015 [24]	15	6	40	55	111	20	40	100	111	23	40	110	111	26	40	111	111	26	40	111	111	25	40	111	111	N/A	N/A	N/A	N/A	N/A	N/A	N/A	N/A
Ito 2015 [23]	24.8	0	14	31	56	9	14	51	56	N/A	N/A	N/A	N/A	N/A	N/A	N/A	N/A	N/A	N/A	N/A	N/A	N/A	N/A	N/A	N/A	N/A	N/A	N/A	N/A	N/A	N/A	N/A	N/A
Kinoshita 2015 [22]	60	4	34	15	23	17	34	23	23	N/A	N/A	N/A	N/A	N/A	N/A	N/A	N/A	N/A	N/A	N/A	N/A	N/A	N/A	N/A	N/A	N/A	N/A	N/A	N/A	N/A	N/A	N/A	N/A
Li 2015 [21]	N/A	7	25	14	24	N/A	N/A	N/A	N/A	N/A	N/A	N/A	N/A	17	25	24	24	N/A	N/A	N/A	N/A	N/A	N/A	N/A	N/A	N/A	N/A	N/A	N/A	14	25	7	24
Sato 2017 [8]	20.5	1	33	23	67	12	33	54	67	24	33	67	67	N/A	N/A	N/A	N/A	N/A	N/A	N/A	N/A	N/A	N/A	N/A	N/A	N/A	N/A	N/A	N/A	N/A	N/A	N/A	N/A
Yamada 2016 [20]	12	17	44	19	28	34	44	25	28	40	44	28	28	N/A	N/A	N/A	N/A	N/A	N/A	N/A	N/A	N/A	N/A	N/A	N/A	N/A	N/A	N/A	N/A	N/A	N/A	N/A	N/A
Fukuchi 2018 [19]	11	4	31	49	63	15	31	63	63	N/A	N/A	N/A	N/A	N/A	N/A	N/A	N/A	N/A	N/A	N/A	N/A	N/A	N/A	N/A	N/A	2	31	0	63	22	31	6	63
Ohnuma 2021 [7]	39.3	9	44	14	44	26	44	39	44	36	44	44	44	16	44	32	44	31	44	42	44	N/A	N/A	N/A	N/A	N/A	N/A	N/A	N/A	N/A	N/A	N/A	N/A
Li 2021 [18]	14.1	16	40	24	40	40	40	40	40	N/A	N/A	N/A	N/A	N/A	N/A	N/A	N/A	N/A	N/A	N/A	N/A	N/A	N/A	N/A	N/A	7	40	3	40	14	40	11	40
Shinkai 2022 [16]	31.8	7	33	9	19	21	33	18	19	25	33	19	19	N/A	N/A	N/A	N/A	N/A	N/A	N/A	N/A	N/A	N/A	N/A	N/A	1	33	0	19	7	33	4	19
Liang 2023 [15]	16.7	13	42	61	94	39	42	94	94	N/A	N/A	N/A	N/A	15	42	76	94	39	42	94	94	N/A	N/A	N/A	N/A	N/A	N/A	N/A	N/A	N/A	N/A	N/A	N/A

*N/A* not available, *S* surgery, *NS* non- surgery, *CR* complete response, *PR* partial responses

**Table 4:** The characteristics and results of the interventions used in each study

Author + year	SD				PD				Neutropenia				Anemia				Nausea				Diarrhea				obstruction			
Group	S		NS		S		NS		S		NS		S		NS		S		NS		S		NS		S		NS	
Wang 2014 [25]	5	28	12	20	2	28	5	20	N/A	N/A	N/A	N/A	N/A	N/A	N/A	N/A	N/A	N/A	N/A	N/A	N/A	N/A	N/A	N/A	N/A	N/A	N/A	N/A
Fukuchi 2015 [24]	N/A	N/A	N/A	N/A	N/A	N/A	N/A	N/A	N/A	N/A	N/A	N/A	N/A	N/A	N/A	N/A	N/A	N/A	N/A	N/A	N/A	N/A	N/A	N/A	N/A	N/A	N/A	N/A
Ito 2015 [23]	N/A	N/A	N/A	N/A	N/A	N/A	N/A	N/A	N/A	N/A	N/A	N/A	N/A	N/A	N/A	N/A	N/A	N/A	N/A	N/A	N/A	N/A	N/A	N/A	N/A	N/A	N/A	N/A
Kinoshita 2015 [22]	N/A	N/A	N/A	N/A	N/A	N/A	N/A	N/A	N/A	N/A	N/A	N/A	N/A	N/A	N/A	N/A	N/A	N/A	N/A	N/A	N/A	N/A	N/A	N/A	N/A	N/A	N/A	N/A
Li 2015 [21]	8	25	3	24	N/A	N/A	N/A	N/A	5	25	8	24	6	25	10	24	2	25	3	24	5	25	6	24	1	25	1	24
Sato 2017 [8]	N/A	N/A	N/A	N/A	N/A	N/A	N/A	N/A	24	33	51	67	4	33	8	67	8	33	24	67	4	33	10	67	N/A	N/A	N/A	N/A
Yamada 2016 [20]	N/A	N/A	N/A	N/A	N/A	N/A	N/A	N/A	N/A	N/A	N/A	N/A	N/A	N/A	N/A	N/A	N/A	N/A	N/A	N/A	N/A	N/A	N/A	N/A	2	28	2	20
Fukuchi 2018 [19]	7	31	2	63	N/A	N/A	N/A	N/A	N/A	N/A	N/A	N/A	N/A	N/A	N/A	N/A	N/A	N/A	N/A	N/A	N/A	N/A	N/A	N/A	N/A	N/A	N/A	N/A
Ohnuma 2021 [7]	N/A	N/A	N/A	N/A	N/A	N/A	N/A	N/A	N/A	N/A	N/A	N/A	N/A	N/A	N/A	N/A	N/A	N/A	N/A	N/A	N/A	N/A	N/A	N/A	N/A	N/A	N/A	N/A
Li 2021 [18]	13	40	14	40	6	40	12	40	29	40	24	40	N/A	N/A	N/A	N/A	N/A	N/A	N/A	N/A	N/A	N/A	N/A	N/A	N/A	N/A	N/A	N/A
Shinkai 2022 [16]	3	33	0	19	2	33	1	19	7	33	9	19	0	33	5	19	0	33	0	19	0	33	2	19	N/A	N/A	N/A	N/A
Liang 2023 [15]	N/A	N/A	N/A	N/A	N/A	N/A	N/A	N/A	4	42	7	94	11	42	19	94	0	42	3	94	1	42	2	94	N/A	N/A	N/A	N/A

*N/A* not available, *S* surgery, *NS* non- surgery, *SD* stable disease, *PD* progressive disease

### Statistical analysis

Statistical analyses were performed using RevMan 5.4. Dichotomous data were expressed as relative risk (RR) with 95% confidence intervals (CIs). Heterogeneity was evaluated using the I^2^ statistic, with a fixed-effects model used for I^2^ < 50% and a random-effects model for I^2^ > 50%. For I^2^ > 80%, sensitivity analyses were conducted to exclude studies with significant heterogeneity and improve the reliability of results.

## Results

### Primary outcomes

#### 1-Year survival rate

**Fig. 2:**
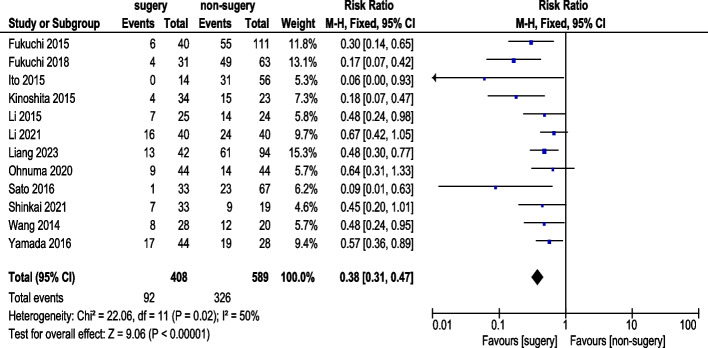
1-Year Survival Rate

Twelve studies reported 1-year OS rate (Fig. 2). A total of 408 AGC patients underwent conversion therapy with surgery, while 589 received chemotherapy alone. Fixed-effects model analysis revealed acceptable heterogeneity. The pooled analysis demonstrated a statistically significant difference in 1-year OS rate (RR: 0.38, 95% CI: 0.31–0.47).

#### 1-Year progression-free survival rate

**Fig. 3:**
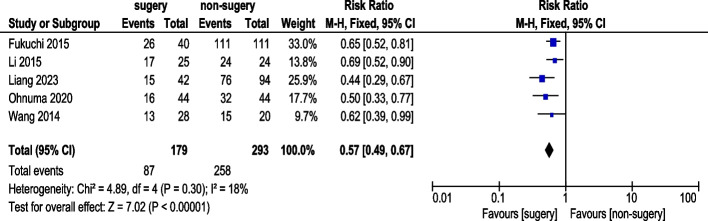
1-Year Progression-Free Survival Rate

Five studies reported 1-year PFS rate (Fig. 3). A total of 179 AGC patients underwent conversion surgery, while 293 received chemotherapy alone. Fixed-effects model analysis showed acceptable heterogeneity, and pooled results revealed a statistically significant difference in 1-year PFS rate (RR: 0.57, 95% CI: 0.49–0.99).

#### 3-Year survival rate

**Fig. 4:**
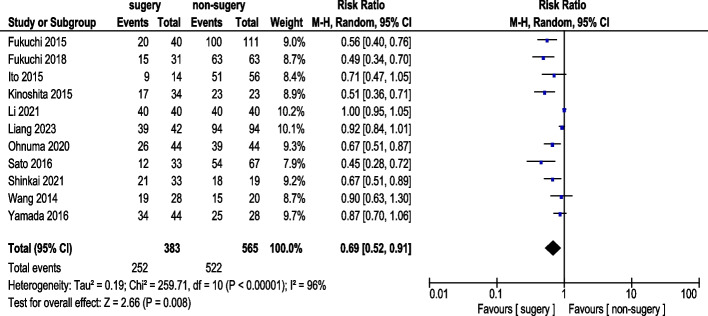
3-Year Survival Rate (pre-sensitivity analysis)

**Fig. 5:**
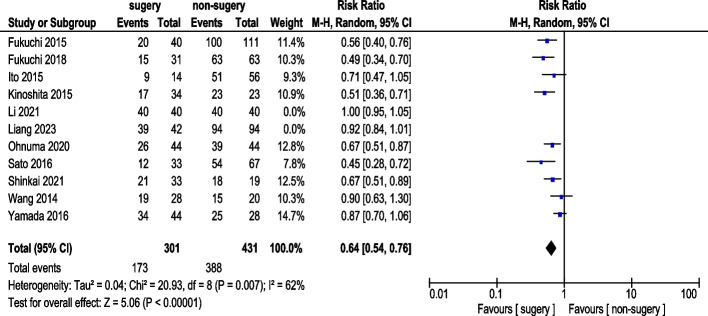
3-Year Survival Rate (post-sensitivity analysis)

Eleven studies reported 3-year OS rate (Fig. 4). A total of 383 AGC patients underwent conversion surgery, while 626 received chemotherapy alone. Random-effects model analysis revealed significant heterogeneity. The pooled analysis demonstrated a statistically significant difference in 3-year OS rate (RR: 0.69, 95% CI: 0.52–0.91). Sensitivity analyses excluding Li (2021) and Liang (2023) reduced heterogeneity, and pooled results from nine studies showed a statistically significant improvement in 3-year OS rate (RR: 0.64, 95% CI: 0.54–0.76) (Fig. 5). Exclusion of Li (2021) and Liang (2023) reduced heterogeneity from I^2^ = 96% to 62%. These studies differed methodologically: Li (2021) included patients with adjuvant chemotherapy with unknown regimen while Liang (2023) used immunotherapy-based conversion therapy, potentially confounding survival outcomes.

#### 3-Year progression-free survival rate

**Fig. 6:**
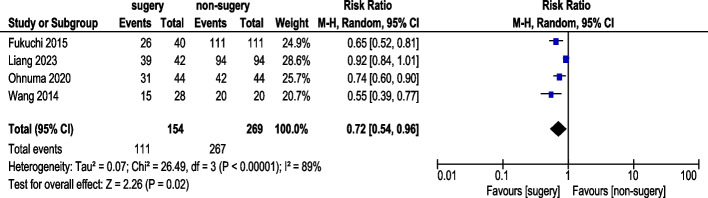
3-Year Progression-Free Survival Rate (pre-sensitivity analysis)

**Fig. 7:**
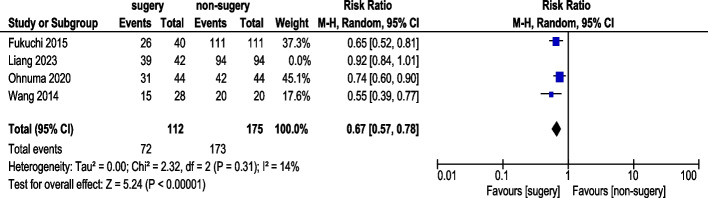
3-Year Progression-Free Survival Rate (post-sensitivity analysis)

Four studies reported 3-year PFS rate (Fig. 6). A total of 154 AGC patients underwent conversion surgery, while 269 received chemotherapy alone. Random-effects model analysis revealed significant heterogeneity. The pooled analysis demonstrated a statistically significant difference in 3-year PFS rate (RR: 0.72, 95% CI: 0.54–0.96). After excluding Liang (2023) in sensitivity analysis, three studies demonstrated a statistically significant improvement in 3-year PFS rate (RR: 0.67, 95% CI: 0.57–0.78) (Fig. 7). Exclusion of Li (2021) reduced heterogeneity from I^2^ = 89% to 14%. This study differed methodologically: Li (2021) included patients with adjuvant chemotherapy with unknown regimen.

#### 5-Year survival rate

**Fig. 8:**
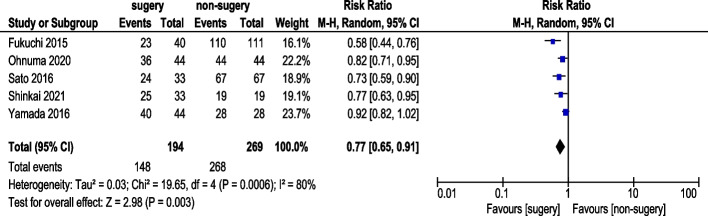
5-Year Survival Rate

Five studies reported 5-year OS rate (Fig. 8). A total of 194 AGC patients underwent conversion surgery, while 269 received chemotherapy alone. Random-effects model analysis revealed acceptable heterogeneity, and pooled results indicated a statistically significant improvement in 5-year OS rate (RR: 0.77, 95% CI: 0.65–0.91).

### Secondary outcomes

#### Complete Response (CR) rate

**Fig. 9:**
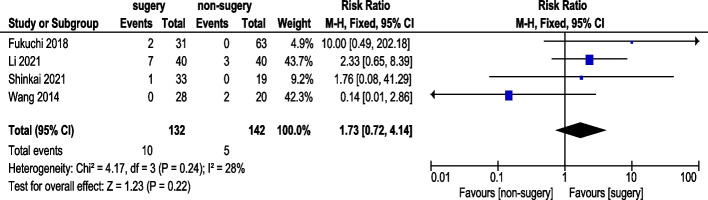
Complete Response (CR) Rate

Five studies reported CR rates (Fig. 9). A total of 132 AGC patients underwent conversion surgery, while 142 received chemotherapy alone. Random-effects model analysis revealed no statistically significant difference in CR rates (RR: 1.73, 95% CI: 0.72–4.14).

#### Partial Response (PR) rate

**Fig. 10:**
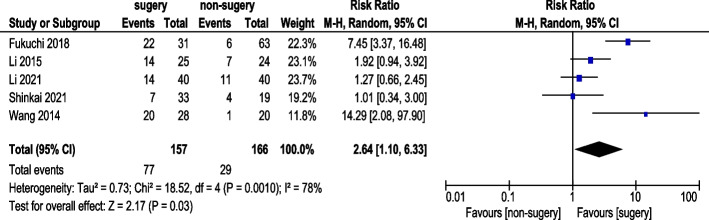
Partial Response (PR) Rate

Five studies reported PR rates (Fig. 10). A total of 157 AGC patients underwent conversion surgery, while 166 received chemotherapy alone. Random-effects model analysis revealed a statistically significant improvement in PR rates (RR: 2.64, 95% CI: 1.10–6.33).

#### Stable disease (SD) rate

**Fig. 11:**
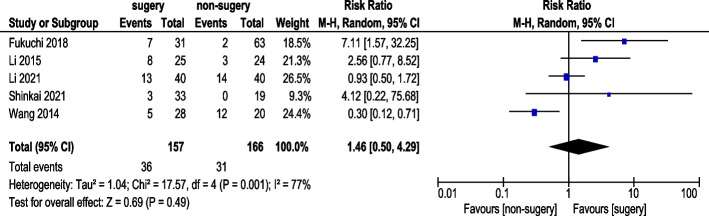
Stable disease (SD) Rate

Five studies reported SD rates (Fig. 11). A total of 157 AGC patients underwent conversion therapy followed by surgery, while 166 patients received chemotherapy alone. Under a random-effects model, overall heterogeneity was acceptable. The analysis indicated no statistically significant difference in SD rates (RR 1.46, 95% CI: 0.50–4.29).

#### Progressive disease (PD) rate

**Fig. 12:**
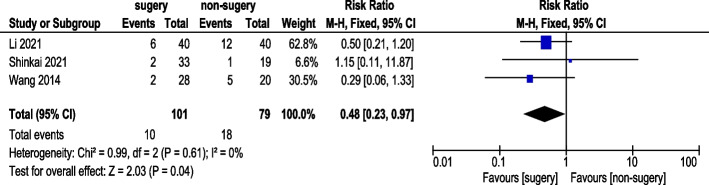
Progressive disease (PD) Rate

Three studies reported PD rates (Fig. 12). A total of 101 AGC patients underwent conversion therapy followed by surgery, while 79 patients received chemotherapy alone. Using a fixed-effects model, the overall heterogeneity was acceptable. The analysis indicated a statistically significant difference in PD rates (RR 0.48, 95% CI: 0.23–0.97).

### Adverse event rates

#### Neutropenia incidence

**Fig. 13:**
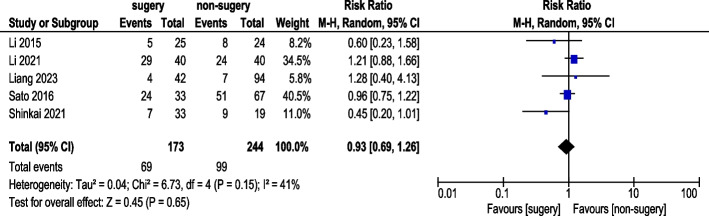
Neutropenia Incidence

Five studies reported neutropenia incidence (Fig. 13). A total of 173 AGC patients underwent conversion therapy followed by surgery, while 244 patients received chemotherapy alone. Under a random-effects model, overall heterogeneity was acceptable. The analysis showed no statistically significant difference in neutropenia incidence (RR 0.93, 95% CI: 0.69–1.26).

#### Anemia incidence

**Fig. 14:**
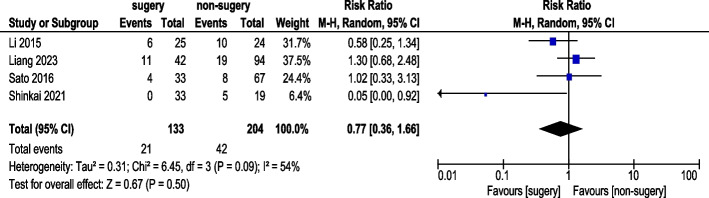
Anemia Incidence

Four studies reported anemia incidence (Fig. 14). A total of 133 AGC patients underwent conversion therapy followed by surgery, while 204 patients received chemotherapy alone. Under a random-effects model, overall heterogeneity was acceptable. The analysis showed no statistically significant difference in anemia incidence (RR 0.77, 95% CI: 0.36–1.66).

#### Nausea incidence

**Fig. 15:**
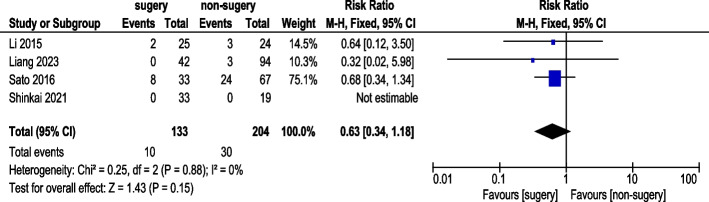
Nausea Incidence

Four studies reported nausea incidence (Fig. 15). A total of 133 AGC patients underwent conversion therapy followed by surgery, while 204 patients received chemotherapy alone. Using a fixed-effects model, overall heterogeneity was acceptable. The analysis showed no statistically significant difference in nausea incidence (RR 0.63, 95% CI: 0.34–1.34).

#### Diarrhea incidence

**Fig. 16:**
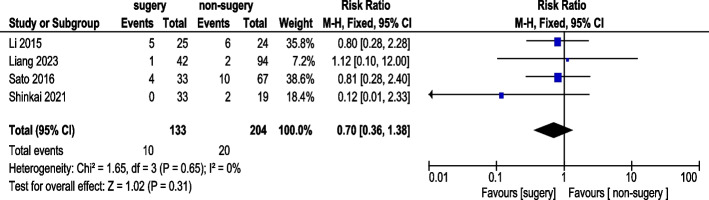
Diarrhea Incidence

Four studies reported diarrhea incidence (Fig. 16). A total of 133 AGC patients underwent conversion therapy followed by surgery, while 204 patients received chemotherapy alone. Using a fixed-effects model, overall heterogeneity was acceptable. The analysis showed no statistically significant difference in diarrhea incidence (RR 0.70, 95% CI: 0.36–1.38).

#### Bowel obstruction incidence

**Fig. 17:**
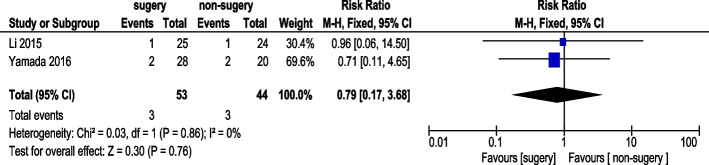
Bowel Obstruction Incidence

Two studies reported bowel obstruction incidence (Fig. 17). A total of 53 AGC patients underwent conversion therapy followed by surgery, while 44 patients received chemotherapy alone. Using a fixed-effects model, overall heterogeneity was acceptable. The analysis showed no statistically significant difference in bowel obstruction incidence (RR 0.79, 95% CI: 0.17–3.68).

## Discussion

Current management of advanced gastric cancer (AGC) with unresectable factors lacks standardized protocols regarding chemotherapy regimens, surgical approaches, and the influence of metastatic burden on therapeutic strategies [26–28]. While common regimens such as SP, DS, DOS, XELOX, SOX, and FOLFOX are utilized, their safety and efficacy vary significantly, as highlighted by Liu et al. [29]. For II/III-stage gastric cancer, standard adjuvant chemotherapy regimens include S-1, capecitabine + oxaliplatin, and S-1 + docetaxel [13, 30–32]. In stage IV, palliative surgery combined with chemotherapy fails to enhance long-term survival [33].; however, the AIO-FLOT3 trial demonstrated markedly prolonged median survival (31.3 vs. 15.9 months) with R0 resection post-FLOT therapy [34]. For peritoneal metastases, cytoreductive surgery (CRS) with hyperthermic intraperitoneal chemotherapy (HIPEC) benefits patients with a peritoneal cancer index (PCI) ≤ 6, whereas systemic chemotherapy plus neoadjuvant intraperitoneal chemotherapy (NIPEC) is preferred for PCI > 6 [35]. Despite emerging evidence supporting postoperative chemotherapy after CRS in stage IV patients [2, 36, 37]. Further high-quality clinical trials are needed to confirm the effectiveness and appropriate chemotherapy cycles for patients undergoing conversion surgery.

In this meta-analysis, patients in the surgical group had higher survival rates compared to those in the non-surgical group, for several reasons. First, the severity of each patient's condition differs, particularly in terms of the number of unresectable clinical factors. Most patients in the surgical group responded to chemotherapy, whereas non-surgical patients typically did not respond. The survival benefit observed in the surgical group must be interpreted cautiously. Patients undergoing conversion surgery were inherently selected based on favorable responses to chemotherapy, suggesting their tumors may have had less aggressive biology. Thus, the observed association between surgery and improved outcomes may partly reflect this selection bias rather than a direct causal effect of surgery. While conversion surgery appears beneficial for select responders, clinical decisions must account for tumor biology. Universal application of this approach is not yet supported by high-level evidence. Additionally, the degree of adverse effects from conversion therapy is a key issue. The results of this meta-analysis indicate that conversion therapy is feasible and safe, and most advanced cancer patients can tolerate chemotherapy toxicity and complete the chemotherapy regimen. The lack of CR improvement despite higher PR rates may reflect tumor biology: partial responders may benefit more from cytoreduction, while complete responders could have micrometastases undetected by imaging, leading to recurrence post-surgery. While conversion therapy can significantly increase the rate of conversion surgery, there are still challenges in performing conversion surgery. The primary reason is that patients with advanced cancer often have poor physical status, which leads to a reduction in the dose of chemotherapy drugs. This makes it difficult to convert patients from an unresectable to a resectable state. Furthermore, determining which patients are suitable for conversion surgery and assessing the optimal timing for the surgery is another challenge. The best timing for surgery is when the tumor shows the best response to chemotherapy, before the tumor becomes resistant to chemotherapy drugs [38]. According to previous studies, the duration of conversion therapy for AGC patients depends on the response to chemotherapy, and the conversion treatment is typically performed 5–6 weeks after the final chemotherapy cycle [8]. Moreover, previous research has indicated that the surgical duration, blood loss, and postoperative hospital stay are all within acceptable ranges [39]. Similarly, this meta-analysis indicates that the incidence of postoperative adverse reactions is also acceptable. According to previous clinical trials, only curative resections (R0 resection) are associated with long-term survival, while patients who undergo non-curative tumor resection have very poor prognosis [8, 14, 22, 24, 40–42]. Likewise, the results of this meta-analysis suggest that patients who undergo therapeutic resection perform better than those who receive non-therapeutic reimplantation. Therefore, conversion surgery should aim for R0 resection.

Surgically, laparoscopic/robotic gastrectomy with D2 lymphadenectomy is increasingly adopted for AGC [43], yet minimally invasive CRS remains unvalidated. Debates persist regarding optimal surgical approach (open vs. minimally invasive), lymphadenectomy extent, and the utility of omentectomy/splenectomy. Splenectomy should be avoided in non-greater curvature tumors due to elevated morbidity without survival benefit [44], while omentectomy’s prognostic value remains unproven [45].

In conclusion, conversion therapy represents a feasible pathway to R0 resection in select AGC patients, though standardized protocols for patient selection, chemotherapy duration, and surgical techniques await further investigation. Future randomized controlled trials should adopt designs where chemotherapy responders are randomized to surgery versus continued non-surgical therapy. Such trials would directly evaluate the additive value of surgery, independent of baseline prognostic differences.

### Limitations

Firstly, our study subjects were patients with clinical stage III-IV advanced gastric cancer (AGC). The included studies were high-quality observational cohort studies, but no randomized controlled trials were available. The absence of RCTs introduces selection bias, as patients selected for surgery may have better baseline prognoses. We conducted sensitivity analyses excluding studies with NOS < 7. The selection bias inherent in observational cohort studies may still impact the accuracy of the results. Secondly, although the studies included were predominantly from Asian regions, the applicability of these findings to Western populations remains unclear. Additionally, many studies did not provide a detailed analysis of the role of adjuvant chemotherapy and its impact within multimodal treatment approaches. Thirdly, regarding the choice of drugs in conversion therapy regimens, insufficient research exists to evaluate the efficacy of different drugs. Our meta-analysis did not analyze the specific effects of various drugs used in conversion therapy on patient prognosis. Fourthly, the safety and feasibility of performing conversion surgery using minimally invasive methods have not yet been established. The indications for conversion surgery approaches (open, laparoscopic, or robotic) and the extent of lymphadenectomy remain controversial. Fifthly, the absence of randomized data means residual confounding factors, such as unmeasured tumor biology or patient fitness, may influence outcomes. While sensitivity analyses adjusted for methodological heterogeneity, they cannot fully address the fundamental prognostic imbalance between groups.

## Conclusion

Our findings suggest that treatment strategies for AGC patients with initially unresectable factors should consider conversion therapy followed by surgery. The results of this meta-analysis indicate that AGC patients who underwent conversion therapy followed by surgery had significantly improved survival rates and progression-free survival compared to those who received conversion therapy alone without surgery. However, the efficacy and safety of conversion surgery still require support from higher-level evidence. Further randomized controlled trials are necessary to validate these findings.

## Supplementary Information

Supplementary Material 1.

## Data Availability

The data that support the findings of this study are available from the PubMed, Embase, and Web of Science.

## Abbreviations

AGC: Advanced gastric cancer
CS: Conversion surgery
OS: Overall survival
PFS: Progression-free survival
CR: Complete response
PR: Partial response
SD: Stable disease
PD: Progressive disease
PCI: Peritoneal metastasis index
CRS: Cytoreductive surgery
NIPEC: Neoadjuvant intraperitoneal chemotherapy
