# Supplementary material for: Comparison of outcomes between surgery and non-surgery after conversion therapy for advanced gastric cancer with unresectable factors: a systematic review and meta-analysis
Source: BMC Gastroenterol. 2025 May 14;25:371. doi: 10.1186/s12876-025-03969-x (PMC12080213; doi:10.1186/s12876-025-03969-x)
Supplement: Supplementary file 1 — Supplementary Material 1. [file 12876_2025_3969_MOESM1_ESM.docx]

PubMed:

("gastric cancer"[title] OR "stomach neoplasms"[title] OR "cancer of stomach") AND ("surgery"[title] OR "operation"[title]) AND ("chemotherapy"[title])

Filters: 2014/01/01–2024/11/30, English language

Embase:

('gastric cancer':ti OR 'gastric carcinoma':ti OR 'cancer of stomach':ti) AND (surgery:ti OR operation:ti) AND ('chemotherapy':ti)

Filters: Publication date 2014–2024

Web of Science:

TI=("gastric cancer" OR "stomach neoplasms" OR "cancer of stomach") AND TI=("surgery" OR " operation") AND TI=(" chemotherapy")
